# Quantitative evaluation of cardiac magnetic resonance feature tracking (CMR-FT) derived strain in patients with ST-elevation myocardial infarction

**DOI:** 10.12669/pjms.39.3.7571

**Published:** 2023

**Authors:** Sahrai Saeed, Erlend Eriksen, Terje H. Larsen

**Affiliations:** 1 Sahrai Saeed, Department of Heart Disease, Haukeland University Hospital, Bergen, Norway; 2 Erlend Eriksen, Department of Heart Disease, Haukeland University Hospital, Bergen, Norway; 3 Terje H. Larsen Department of Biomedicine, University of Bergen, Norway

Left ventricular (LV) myocardial strain is a direct measure of myocardial deformation and an excellent method for assessment of systolic LV function. This measure overcomes some of the inherent limitations of the conventional LV ejection fraction (EF). Strain is reported as percentage lengthening and shortening, where systolic shortening results in negative and lengthening in positive strains.^1^ Myocardial strain was initially derived from tissue Doppler imaging nearly 2.5 decades ago.^2^ However, this method turned out to have limited clinical reproducibility, was angle-dependent and had significant noise. The field was further developed and speckle tracking echocardiography (STE) soon emerged as a more accurate and user-friendly technique.

Strain imaging allows assessment of global LV function as reflected by global longitudinal (GLS) and global circumferential strain (GCS). GLS is widely used and has important clinical and prognostic implications in a number of cardiovascular diseases including hypertension, stroke, aortic stenosis and coronary artery disease, or in patients undergoing chemotherapy for cancer.^3^-^7^ It has been shown that in aortic stenosis GLS if often impaired at early stages when EF is still normal. The impairment may be either global or regional. Furthermore, in patients with aortic stenosis, hypertension and increased arterial stiffness are highly prevalent^8^-^10^, which both affect myocardial performance as reflected by impaired LV strain.

Strain imaging also allows assessment of regional LV dysfunction/ischemia when segmental strain is impaired (less negative value) or show other pathological patterns such early systolic lengthening or post-systolic shortening of both left and right ventricular (RV) myocardium.^11^-^14^ However, STE imaging is also subject to all the inherent limitations of ultrasound such as image quality and operator dependency, as well as the challenges with assessing strain of cardiac chambers with thin walls such as the atria and the RV free wall. Hence, although most evidence and clinical experience regarding strain measures have been obtained by echocardiography, the clinical applicability of strain imaging has now evolved into the field of cardiac magnetic resonance (CMR).^15^-^17^ There is a good correlation between myocardial strain parameters measured with STE and CMR feature-tracking (CMR-FT). However, GLS appears to be more accurate with STE, while GCS shows a better reproducibility when measured by CMR-FT. Nonetheless, similar to echocardiography, differences between various CMR-FT software may result in differing values.^18^

In a recent article published in the journal (PJMS), Jun Ye et al.^19^ investigated the clinical significance of CMR-FT in 100 patients with acute myocardial infarction. EF was comparable in STEMI and NSTEMI patients but radial, circumferential and longitudinal strain was more impaired in STEMI patients. The correlation between longitudinal strain and late gadolinium enhancement (LGE) was stronger (-0.450, p<0.001) than circumferential (-0.313, p=0.002) and radial strain (-0.263, p=0.008), while circumferential strain had the greatest statistical area under the curve (AUC) (0.866) for diagnostic value of STEMI compared with longitudinal (0.788) and radial strain (0.677, all p<0.001). These findings are clinically relevant and make a useful addition to the existing literature on the subject. However, as acknowledged by the authors, the study was retrospective and had a small sample size with some additional methodological limitations.

In a study of 323 patients who underwent CMR one week after STEMI, Gavara et al.^15^ showed that CMR-FT provided prognostic information after STEMI but did not significantly improve risk reclassification beyond traditional CMR indexes.^15^ Apart from patients with coronary artery disease, GLS derived from CMR-FT has also been shown to be a more sensitive functional marker in cardiomyopathies, for example Fabry cardiomyopathy. In a study by Vijapurapu and colleagues, CMR-FT was able to identify myocardial mechanical changes in the early stages of Fabry cardiomyopathy.^20^

Hence, although CMR-FT seems to be a promising technique for assessing global and segmental myocardial strain both in patients with STEMI and cardiomyopathies, further well-designed and prospective research studies in different clinical settings with sufficient power that include both CMR-FT (2D and 3D) and STE, are needed to validate strain values measured from CMR-FT images so the exact role of CMR-FT beyond routinely used CMR indexes are documented - before the data can be used in the clinical decision-making process.

## Author Contributions:

**SS** wrote the first draft of the article which was revised by **EE and THL.**

All authors approved the final submission.
